# Electron Microscopy in Discovery of Novel and Emerging Viruses from the Collection of the World Reference Center for Emerging Viruses and Arboviruses (WRCEVA)

**DOI:** 10.3390/v11050477

**Published:** 2019-05-25

**Authors:** Vsevolod L. Popov, Robert B. Tesh, Scott C. Weaver, Nikos Vasilakis

**Affiliations:** 1 Department of Pathology, University of Texas Medical Branch, 301 University Blvd, Galveston, TX 77555, USA; rtesh@utmb.edu; 2 Center for Biodefense and Emerging Infectious Diseases, University of Texas Medical Branch, 301 University Blvd, Galveston, TX 77555, USA; sweaver@utmb.edu; 3 Institute for Human Infection and Immunity, University of Texas Medical Branch, 301 University Blvd, Galveston, TX 77555, USA; 4 Center for Tropical Diseases, University of Texas Medical Branch, 301 University Blvd, Galveston, TX 77555, USA; 5 World Reference Center for Emerging Viruses and Arboviruses, University of Texas Medical Branch, 301 University Blvd, Galveston, TX 77555, USA; 6 Department of Microbiology and Immunology, University of Texas Medical Branch, 301 University Blvd, Galveston, TX 77555, USA

**Keywords:** virus ultrastructure, virus discovery, electron microscopy, virus taxonomy

## Abstract

Since the beginning of modern virology in the 1950s, transmission electron microscopy (TEM) has been an important and widely used technique for discovery, identification and characterization of new viruses. Using TEM, viruses can be differentiated by their ultrastructure: shape, size, intracellular location and for some viruses, by the ultrastructural cytopathic effects and/or specific structures forming in the host cell during their replication. Ultrastructural characteristics are usually sufficient for the identification of a virus to the family level. In this review, we summarize 25 years of experience in identification of novel viruses from the collection of the World Reference Center for Emerging Viruses and Arboviruses (WRCEVA).

Since the beginning of modern virology in the 1950s, transmission electron microscopy (TEM) has been one of the most important and widely used techniques for identification and characterization of new viruses. Two TEM techniques are generally used for this purpose: negative staining on an electron microscopic grid coated with a support film, or (ultra) thin-section TEM of infected cells, fixed, pelleted, dehydrated and embedded in epoxy plastic. Negative staining can be conducted on highly concentrated suspensions of purified virus or on cell culture supernatants. For some viruses, TEM can be conducted on contents of skin lesions (e.g., poxviruses and herpesviruses) or on concentrated stool material (rotaviruses and noroviruses). For successful detection of viruses in ultrathin sections of infected cells, at least 70% of cells must be infected, so either high multiplicity of infection (MOI) or rapid virus multiplication is required.

Viruses can be differentiated by their specific morphology (ultrastructure): shape, size, intracellular location, or from the ultrastructural cytopathic effects and specific structures forming in the host cell during virus replication. Usually, ultrastructural characteristics are sufficient for the identification of a virus to the level of a family. In certain cases, confirmation can be obtained by immuno-EM performed either on virus suspensions before negative staining or on ultrathin sections. This requires virus-specific primary antibodies, which may not available in case of a novel virus. For on-section immuno-EM, OsO_4_ post-fixation must be omitted and the partially dehydrated sample must be embedded in a water-miscible acrylic plastic, usually LR White (Electron Microscopy Services, Hatfield, PA).

The ultrastructure of most common viruses is well documented in good atlases and book chapters [1,2,3,4,5,6,7,8,9,10,11,12] and many classical publications of the 1960s, ‘70s and ‘80s. Several excellent reviews were recently published on use of TEM in detection and identification of viruses [13,14,15,16]. We describe here some examples of the use of EM to assist in the probable taxonomic assignment of novel viruses in the WRCEVA collection.

The WRCEVA serves as a virus reference center for the world. Any zoonotic virus suspected of being biologically transmitted by arthropods or vertebrates is accepted for identification and characterization. A repository collection of more than 9000 virus strains is maintained with complementary sera and diagnostic antigens. In addition to arthropod-transmitted viruses, a number of other zoonotic vertebrate viruses (i.e., poxviruses, paramyxoviruses, orthomyxoviruses, herpesviruses, coronaviruses, hantaviruses, arenaviruses, reoviruses, picornaviruses and rhabdoviruses) together with their respective antigens and antisera are also included in the reference collection. Many of the latter viruses were initially isolated and characterized by arbovirologists, as these agents have occasionally been isolated from clinical samples taken from wild animals or people during arbovirus field studies.

The WRCEVA is a direct outgrowth of the worldwide network of laboratories, first established in the 1950s by the Rockefeller Foundation, to study the role of arthropod-borne viruses in producing human and animal disease and the mechanisms by which these viruses are maintained and transmitted in nature. When this program was initiated at the Rockefeller Foundation Virus Laboratories in New York City in 1951, fewer than 28 arboviruses had been described; and only a few, such as yellow fever, some of the encephalitides, and dengue were known to cause serious disease in human beings. Concurrent with the initiation of the Rockefeller program, the U.S. Army, Navy, Public Health Service and several foreign governments also established arbovirus laboratories and field research programs. This network of field laboratories relied on the Foundation’s central virus reference laboratory in New York until 1964, when the laboratory and some Rockefeller staff were moved to Yale University, and the Yale Arbovirus Research Unit (YARU) was established. In the mid-1995, Drs. Robert Tesh and Robert Shope, at that time directors of the WRCEVA, moved from YARU to the Center for Tropical Diseases (now including the Center for Biodefense and Emerging Infectious Diseases, part of the Institute for Human Infections and Immunity) at the University of Texas Medical Branch (UTMB) in Galveston, and brought the reference collection with them. The decision to establish the Reference Center at UTMB was based in part on the willingness of the University of Texas to provide modern state-of-the-art laboratory equipment and space, including BSL-3 and BSL-4 containment facilities for working with these potentially hazardous agents.

The WRCEVA provides prompt analysis of disease outbreaks as well as identification of new and emerging viruses from field collected samples (e.g., mosquitoes, ticks, birds, sera) provided by agencies around the world. Since its inception, the WRCEVA has been in the forefront of virus discovery and characterization. For example, focusing just on the past 20 years, WRCEVA’s expertise and resources were instrumental in identifying the geographic source of the New York introduction of West Nile virus, and following its westward spread in the U.S. The WRCEVA assisted the Harris County (Houston) Mosquito Control district in the surveillance of West Nile virus in East Texas, testing thousands of mosquito pools and dead birds, an association that continues to this day [17,18,19]. A number of novel avian and mosquito-specific viruses were isolated and characterized during this study. Additionally, the WRCEVA was instrumental in the identification and characterization of several newly discovered tick-borne phleboviruses [20], such as severe fever with fever with thrombocytopenia, Heartland, Uukuniemi group and Bhanja serogroup viruses [21], as well as the new orthomyxoviruses, Upolu, Aransas Bay [22], Sinu [23], and Trinity [24] orthobunyaviruses [25,26,27], nyamiviruses [28], a new reovirus from Cameroon (Fako virus) [29] and Colombia [30], a new paramyxovirus [31], an insect-specific (capable of replication in insects but not in vertebrates) alphavirus [32], a new flavivirus genus [33] and other novel flaviviruses [34,35,36,37] and rhabdoviruses [38,39,40,41,42,43,44,45,46,47]. Lastly, WRCEVA scientists discovered the new family of mesoniviruses [37,48] and the new taxon of the negeviruses [49,50]. In the past decade, the WRCEVA has also been critical in contributing to the surveillance of dengue, chikungunya and Zika viruses during their spread through various parts of the world and identifying their sources and mechanisms of emergence.

In many of these discoveries, transmission electron microscopy (TEM) was essential to determine the taxonomic position (family) of the virus, which will be demonstrated by the following examples.

## 1. Paramyxoviruses

**Figure 1:**
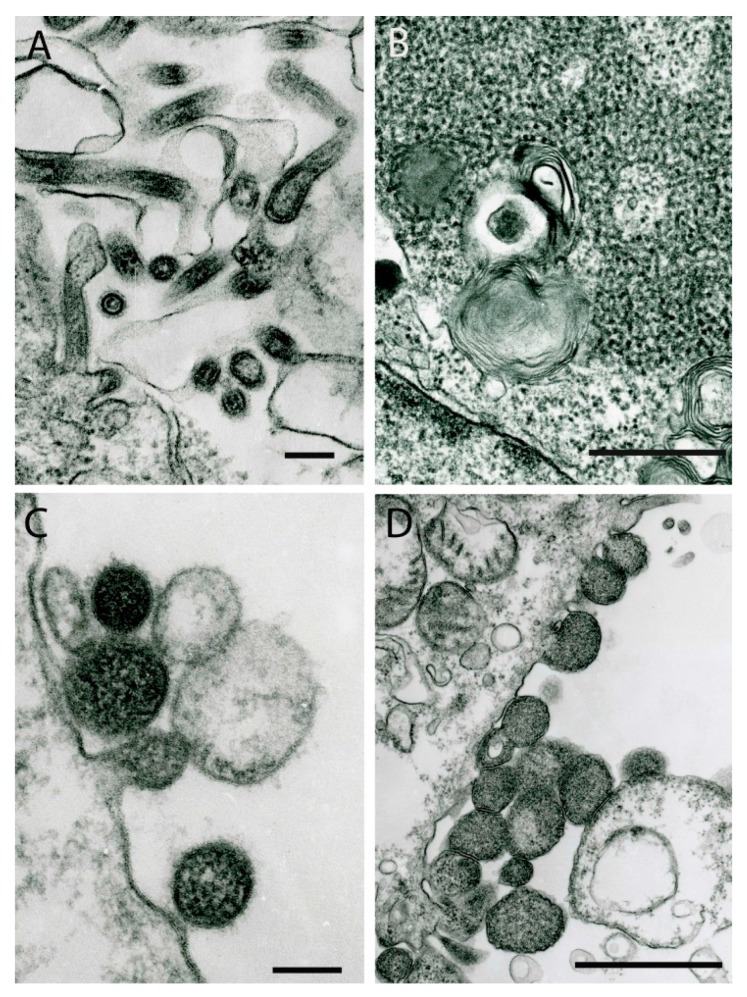
Ultrastructure of viruses of the order *Mononegavirales*. (**A**) JKT-2289 virus (unclassified; probable family *Paramyxoviridae*): virions forming at the cell surface. Bar = 100 nm. (**B**) Cajazeiras virus (strain BeAn 447864) (unclassified; probable family *Paramyxoviridae*): Cytoplasmic inclusion consisting of nucleocapsid nucleoproteins in Vero E6 cell, presented in strands 10–15 nm in diameter. Bar = 0.5 µm. (**C**) Family *Nyamiviridae*: Nyamanini virus virions 100–160 nm in diameter forming at the cell surface. Bar = 100 nm. (**D**) Family *Nyamiviridae*: Sierra Nevada virus virions of variable sizes (300–750 nm in cross-section) forming at the surface of a Vero cell. Bar = 1 µm.

Paramyxoviruses form at the cell surface using plasmalemma as their envelope. Usually they are pleomorphic or spherical in shape, varying in size from 100 nm to several hundred nanometers, but they can also be finger-like or filamentous (Figure 1A). Filamentous virions are 55 nm to 85 nm in diameter and can reach up to 1 μm in length. Virions have spikes at the surface ~7–10 nm long, which make their surface look “fuzzy” in ultrathin sections. They contain a helically packed nucleocapsid which in cross-sections looks like tubules ~10 nm in diameter with ~15 nm periodicity. At earlier stages of infection, cells can contain intracytosolic inclusions consisting of nucleocapsid strands 10 nm to 15 nm in diameter (Figure 1B).

## 2. Rhabdoviruses

**Figure 2:**
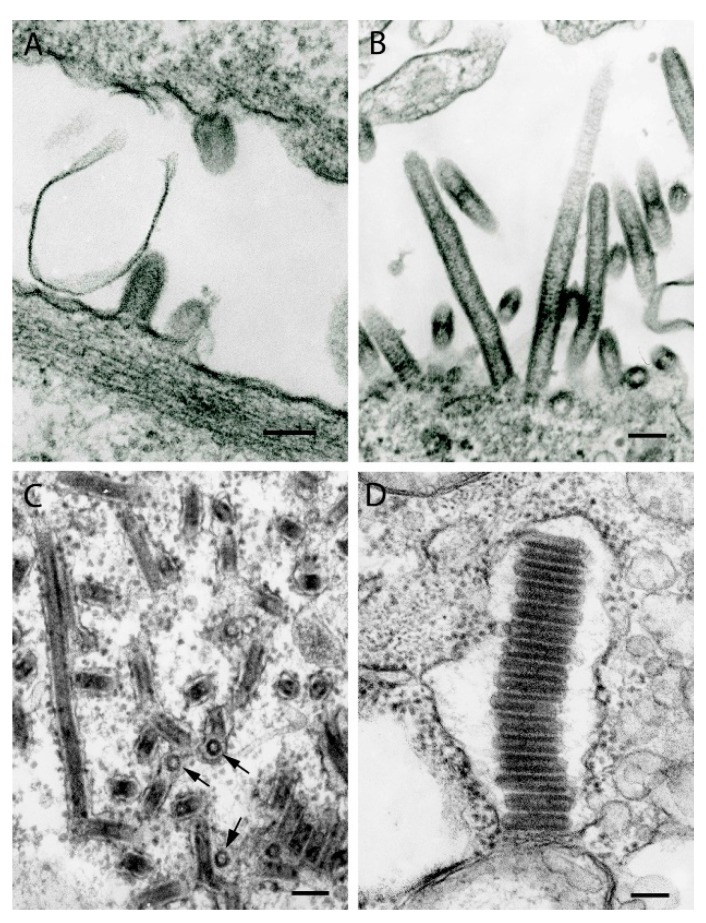
Ultrastructure of viruses of the family *Rhabdoviridae*. (**A**) Harlingen virus, strain PV01-3828 (unclassified; proposed genus *Barhavirus*). Short virions (120–140 nm long, 80 nm in diameter) budding from the surface of Vero E6 cells. (**B**) Sweetwater Branch virus, genus *Tibrovirus*. Long virions (530 nm to 690 nm and up to 900 nm long, 65 nm to 75 nm in diameter) budding from the cell surface. (**C**) Boteke virus, (unclassified; probable genus *Vesiculovirus*). Virions forming inside individual intracytosolic vacuoles in a C6/36 cell. Virions are ~45 nm in diameter and up to 780 nm long. Arrows indicate cross-sections of the virions. (**D**) Niakha virus, strain DakARD88909, genus *Sripuvirus*. A stack of virions 800 nm long in an expanded granular endoplasmic reticulum cistern. Each virion is 55 nm in diameter and 190–215 nm long. Bars = 100 nm.

Rhabdoviruses have characteristic bullet or finger-like morphology with cross-striations 5–7 nm in periodicity, reflecting helical packaging of the nucleocapsid. In cross-sections, they appear ring-like in structure (Figure 2C). The size of virions varies depending on virus, with diameters ranging from 45–80 nm and lengths of 120–200 nm; some virions may be much longer, reaching 900 nm (Figure 2). Defective-interfering particles are shorter and wider, giving them a conical shape. Virions bud from host cell membranes either from the plasmalemma into extracellular space (Figure 2A,B) or into intracellular vacuoles of varying sizes (Figure 2C,D). Inside vacuoles, virions can be packed in stacks of up to 1 µm (Figure 2D). Some rhabdoviruses, especially in animal models, form intracytoplasmic inclusions consisting of nucleoprotein, sometimes with intravacuolar virions [16]. These inclusions correspond to Negri bodies seen in rabies virus infection.

## 3. Nyamiviruses

The family *Nyamiviridae* includes six genera. Nyamanini virus (NYMV), Midway virus, and Sierra Nevada virus (SNVV) were recently assigned to the genus *Nyavirus* [17,18,19]. They are mostly spherical in shape and bud from the cell surface. NYMV virions are 100–160 nm in diameter (Figure 1C), SNVV is larger and more variable in size, its virions measuring 300–750 nm in cross-sections (Figure 1D).

## 4. Orthomyxoviruses

Arboviruses assigned to the family *Orthomyxoviridae* are predominantly tick-borne and recently classified into the genera *Thogotovirus* and *Quaranjavirus* [51]. The genus *Thogotovirus* currently comprises 2 viruses: Thogoto virus (THOV) and Dhori virus (DHOV). Probable members also include Batken virus and its variants, Araguari virus (ARAV) [52], Jos virus (JOSV) [53], Upolu virus (UPOV), Aransas Bay virus (ABV) [22] and the recently isolated Bourbon virus [54].

**Figure 3:**
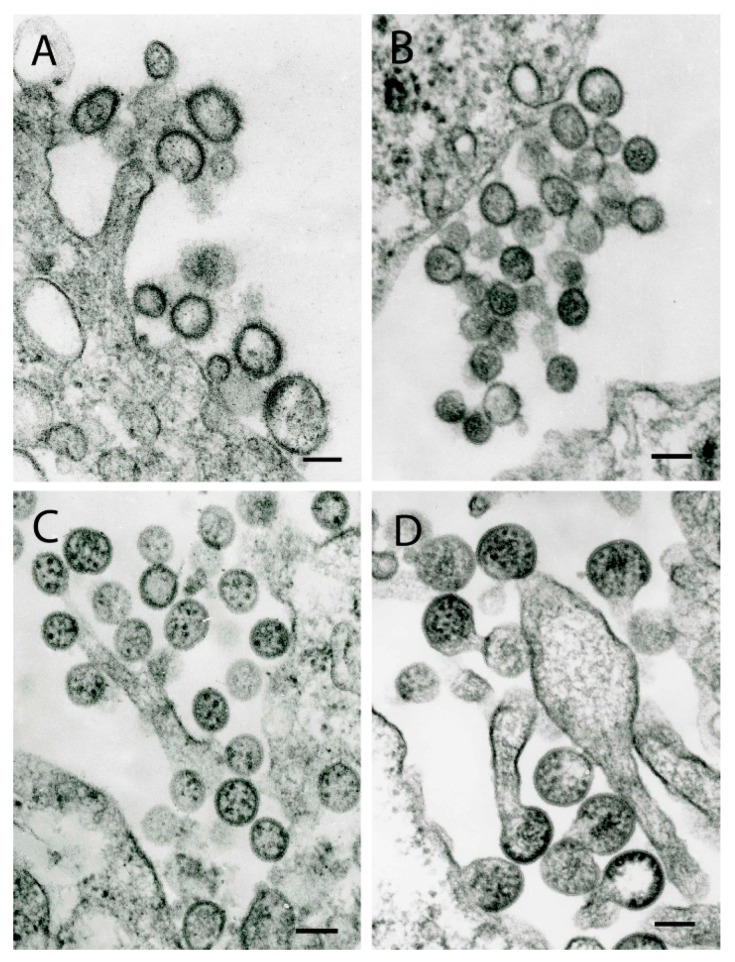
Ultrastructure of tick-borne viruses that are probable members of the family *Orthomyxoviridae*. (**A**) Aransas Bay virus (unclassified; probable genus *Thogotovirus*). Virions 95–200 nm in diameter at the surface of a Vero E6 cell. (**B**) Upolu virus (unclassified; probable genus *Thogotovirus*). A group of virions 75–95 nm in diameter at the surface of a Vero E6 cell. (**C**) Wellfleet Bay virus (unclassified; probable genus *Quaranjavirus*). Virions 95–115 nm in diameter at the surface of a BHK cell. (**D**) Tjulok virus (unclassified; probable genus *Quaranjavirus*). Virions 140–160 nm in diameter forming from the surface of a BHK cell. Bars = 100 nm.

As for other orthomyxoviruses, virions form at the cell surface. THOV and DHOV exhibit significant variations in morphology: they can be pleomorphic, spherical or filamentous, 65 nm in diameter and up to 300 nm long. Other viruses that may be assigned to this genus are mostly spherical but display some polymorphism in sizes: virions of ABV can be 75–200 nm in diameter (Figure 3A), UPOV −75–100 nm (Figure 3B), JOSV −100–140 nm, ARAV −110–40 nm, having spikes at their surface ~7 nm long. Virions of Bourbon virus are mostly pleomorphic, but can be spherical or filamentous [54].

The genus *Quaranjavirus* currently includes two named viruses: Quaranfil virus (QRFV) and Johnston Atoll virus (JAV). Lake Chad virus (LKCV) [51], Wellfleet Bay virus (WFBV), Cygnet River virus [55] and Tjulok (Tyulek or Тюлёк) virus (TLKV) [56] are also probable members of the genus. These viruses are mostly spherical but also display size polymorphism: JAV virions are 140–160 nm in diameter, LKCV – 95–105 nm, WFBV – 85–100 nm (Figure 3C). Most virions of TLKV are ~140 nm in diameter but their size can vary from 105–165 nm (Figure 3D). In ultrathin sections of some virions, up to seven dense granules can be observed representing ribonucleoprotein complexes [55]. The morphology and size of the virions initially led to their misidentification as bunyaviruses or arenaviruses [22,57,58]. Virions mostly form at the cell surface, but with LKCV and TLKV, virions have been observed inside intracytosolic vacuoles with single or two to four virions in a tight vacuole.

## 5. Phleboviruses

**Figure 4:**
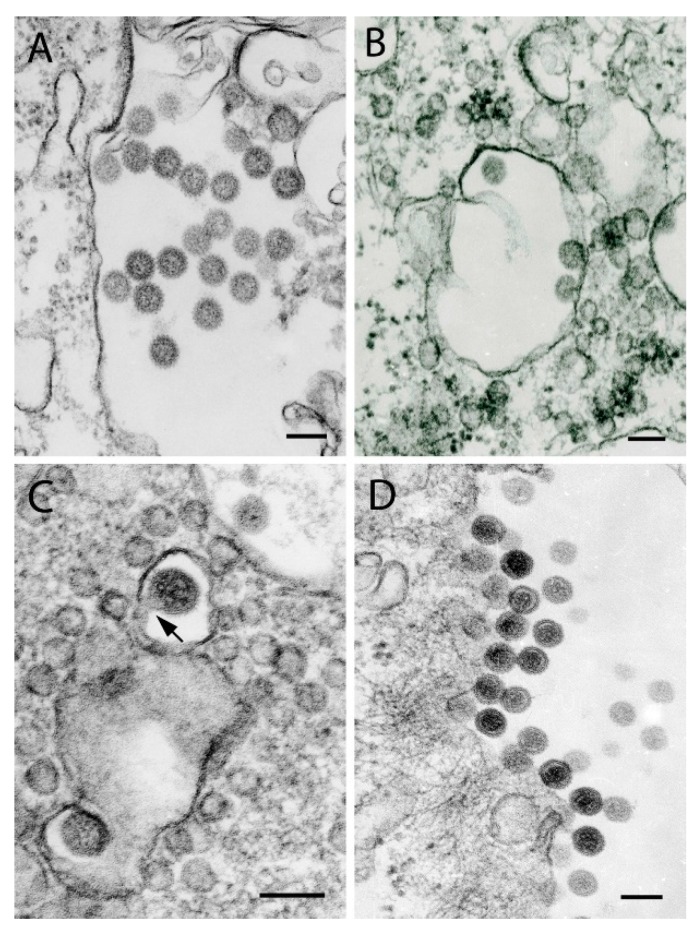
Ultrastructure of tick-borne viruses likely to be assigned to the genus *Phlebovirus*. (**A**) Sunday Canyon virus, Uukuniemi species group. Extracellular virions 75–85 nm in diameter at the cell surface. (**B**) Bhanja virus (strain IbAr 2709), Bhanja group. Three virions 75 nm in diameter in an intracytosolic vacuole. (**C**) Severe fever with thrombocytopenia syndrome (SFTS) virus, SFTSV-Heartland group. Virions 70–80 nm in diameter forming into intracellular vesicles (arrow) in a DH82 cell. (**D**) Heartland virus, SFTSV-Heartland group. Virions 85 nm in diameter at the cell surface of a Vero E6 cell. Bars = 100 nm.

From the largest family of RNA viruses (*Bunyaviridae*), we illustrate the morphology of new and emerging tick-borne viruses that are likely to be assigned to the genus *Phlebovirus*. Phleboviruses are spherical, enveloped viruses 75–95 nm in diameter (Figure 4) sometimes with a dense nucleocapsid core (Figure 4D). Virion envelopes are formed either from the host cell plasmalemma (Figure 4A,D) or from the membrane of small intracytosolic vesicles (Figure 4B,C), usually originating in the Golgi. Envelope glycoproteins are organized into small hollow cylindrical units ~10 nm in diameter arranged in an icosahedral surface lattice revealed in negatively stained virions [59].

## 6. Flaviviruses

**Figure 5:**
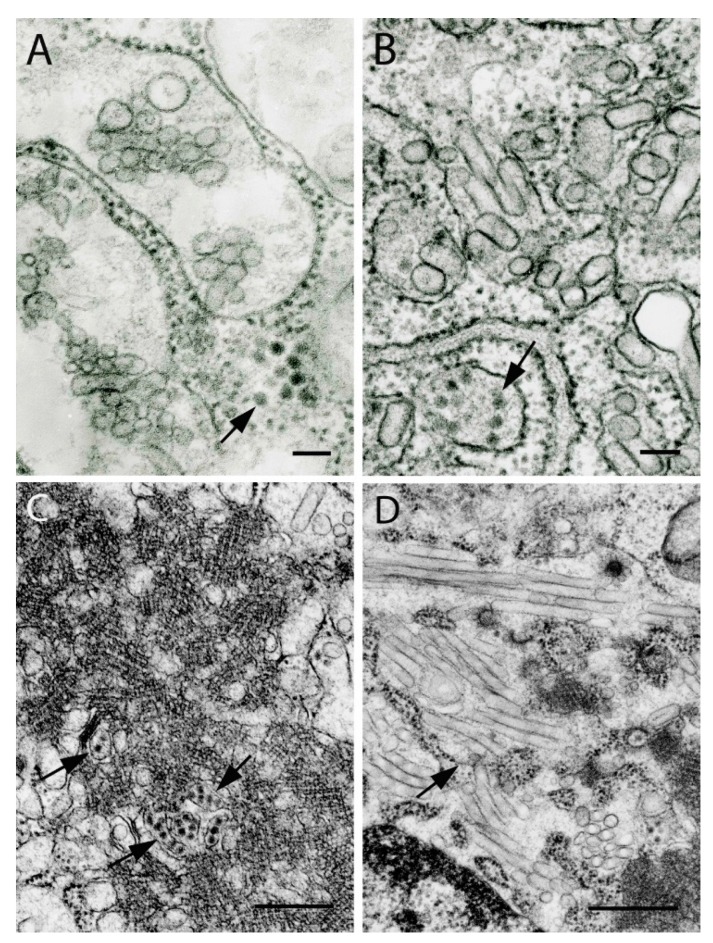
Ultrastructure of flaviviruses (genus *Flavivirus*, family *Flaviviridae*). (**A**) Dengue virus (strain DKE-121). Virions ~40 nm in diameter (arrow) and mostly circular smooth membrane structures (SMS, 25–100 nm in diameter) inside expanded cisterns of granular endoplasmic reticulum (ER). Bar = 100 nm. (**B**) Hu 4578-07 virus (unassigned; probable genus *Flavivirus*). Virions (arrow) and abundant SMS, 50–65 nm in diameter and up to 375 nm long, inside ER. Bar = 100 nm. (**C**) Hu 4578-07 virus. Virions (arrows) inside ER, abundant convoluted membranes. Bar = 0.5 µm. (**D**) Marisma mosquito virus (MMV). Long SMS (up to 2 µm long, ~65 nm in diameter) in expanded ER cisterns (arrows). Bar = 0.5 µm.

Flaviviruses are spherical enveloped viruses ~40 nm in diameter in ultrathin sections, usually with a dense nucleocapsid core. Immature virions are larger in size (~60 nm) and are located inside the cisterns of granular endoplasmic reticulum within infected cells (Figure 5). Replicating flaviviruses induce formation of characteristic intracytoplasmic membraneous structures: convoluted membranes (Figure 5C), paracrystalline arrays and smooth membrane structures (SMS) within usually expanded cisterns of granular endoplasmic reticulum (Figure 5A,B), visible in conventional (epoxy plastic-embedded) ultrathin sections, or vesicle packets in ultrathin cryo-sections [60]. These structures apparently are connected with each other and the membrane system of the cell (endoplasmic reticulum and Golgi) and serve as sites of virus RNA replication and processing thus representing flavivirus replication complexes [61,62]. The presence of even one of these structures can serve as an identifier of an infection of the cell with a flavivirus. SMS can be spherical, 50–65 nm in diameter, or slightly elongated (Figure 5A,B) but in some viruses they can be extremely long, up to 2 μm (Figure 5D).

## 7. Reoviruses

**Figure 6:**
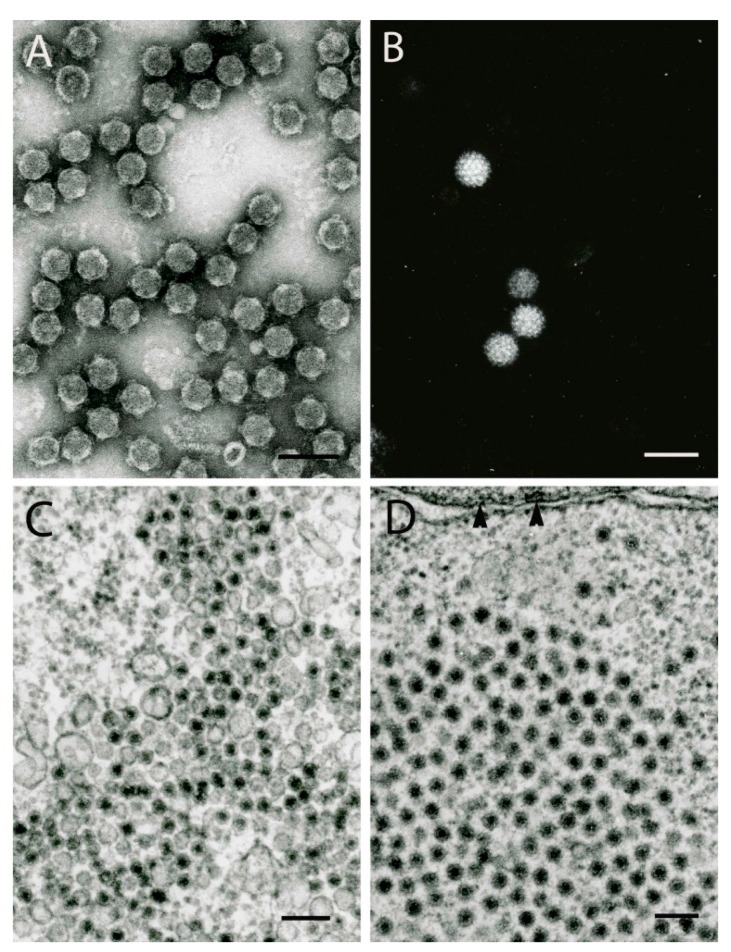
Ultrastructure of viruses of the family *Reoviridae*. (**A**) Negative staining of purified Fako virus (strain CSW77; unassigned probable genus *Dinovernavirus*, subfamily *Spinareovirinae*). Virions 55–60 nm in diameter, each virion image has 5–6 turreted spikes, ~7 nm tall and ~15 nm wide. (**B**) Negative staining of purified Kadipiro virus (strain JKT-7075), genus *Seadornavirus*, subfamily *Sedoreovirinae*. Virions 55–65 nm in diameter, surface subunits ~7 nm in diameter. (**C**) Accumulation of virions of Fako virus (~45 nm in diameter) in an infected C6/36 cell. (**D**) Virions (~45 nm in diameter) of Banna virus (strain JKT-6423), genus *Seadornavirus*, subfamily *Sedoreovirinae*, in the cytoplasm of an infected C6/36 cell. Arrowheads indicate a fragment of the cell nucleus. Bars = 100 nm.

**Figure 7:**
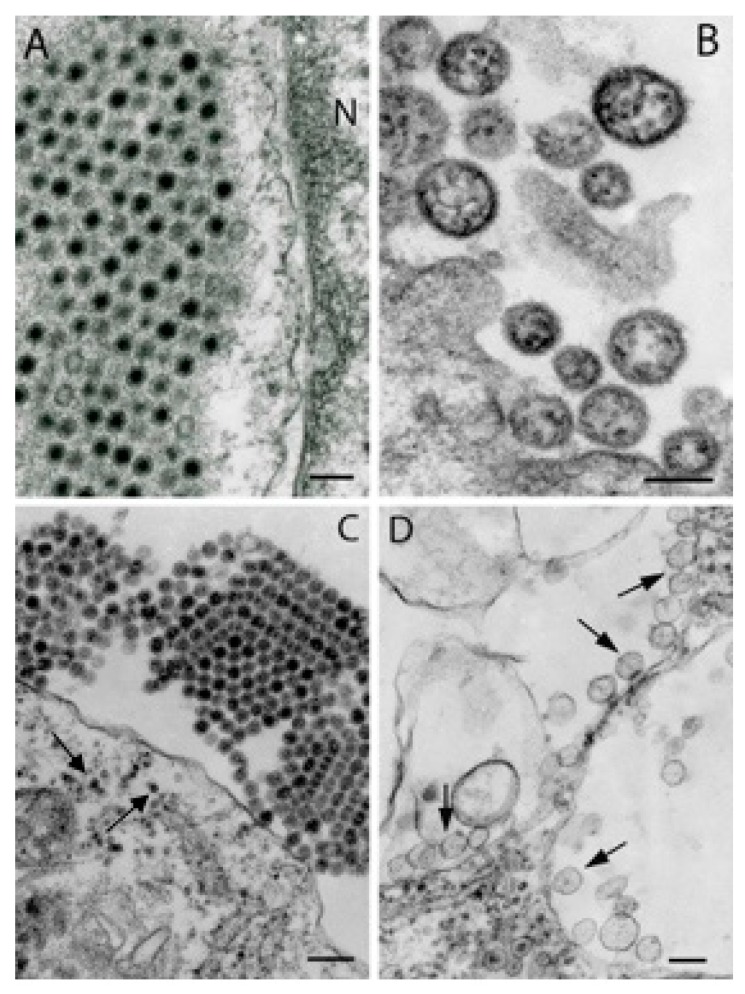
Ultrastructure of viruses recently assigned to or probable members of the families *Reoviridae*, *Arenaviridae*, and *Togaviridae*. (**A**) Fragment of an intracytosolic inclusion of SPU491/84/106 reovirus (unassigned; probable family *Reoviridae*) in a Vero E6 cell. Virions are 55 nm in diameter. N – fragment of the nucleus. (**B**) Virions of SPU-783-85 (unassigned; probable family *Arenaviridae*), 85–130 nm in diameter, at the surface of a Vero 76 cell. (**C**) Paracrystalline package of Trocara virus (genus *Alphavirus*, family *Togaviridae*) virions ~70 nm in diameter at the surface of a C6/36 cell. Nucleocapsid cores (~20 nm in diameter) in the cytoplasm are indicated by arrows. (**D**) Spherules ~75 nm in diameter at the periphery inside of three “cytopathic vacuoles” (arrows) in the cytoplasm of a C6/36 cell infected with Highlands J virus (genus *Alphavirus*, family *Togaviridae*). Bars = 100 nm.

In ultrathin sections of infected cells virions of arthropod-borne reoviruses appear as spherical particles 45–70 nm in diameter with central dense cores (Figure 6C,D and Figure 7A). Family *Reoviridae* includes two subfamilies, *Spinareovirinae* and *Sedoreovirinae*, according to morphology of virion surface which can be visualized in negatively stained preparations of purified viruses. Virions of viruses assigned to the *Spinareovirinae* have short flat spikes or turrets. In Fako virus (unassigned; probable genus *Dinovernavirus*), recently isolated from mosquitoes in Cameroon [29], these turrets are ~7 nm tall and ~15 nm wide (Figure 6A). In the *Sedoreovirinae*, virions are devoid of spikes but can display round surface capsomere subunits ~7 nm in diameter (Figure 6B). Reoviruses reproduce in cytoplasmic inclusions – virus factories, or viroplasms which appear in ultrathin sections as medium density masses composed of fine fibrils and granules and containing virus particles of different stages of maturity and empty shells (Figure 6C,D and Figure 7A). In coltiviruses (genus *Coltivirus*) and orbiviruses (genus *Orbivirus*), virus factories are often associated with randomly distributed fibrils and/or microtubules as demonstrated by F.A. Murphy [58,63].

## 8. Arenaviruses

Arenavirus virions are spherical and pleomorphic with variable sizes, from 80 nm to 200 nm in diameter (Figure 7B). They are surrounded by an envelope ~10 nm thick covered with spikes and typically contain several ribosomes 15 nm to 20 nm in diameter. Virions bud from the cell surface (Figure 7B).

## 9. Alphaviruses

**Figure 8:**
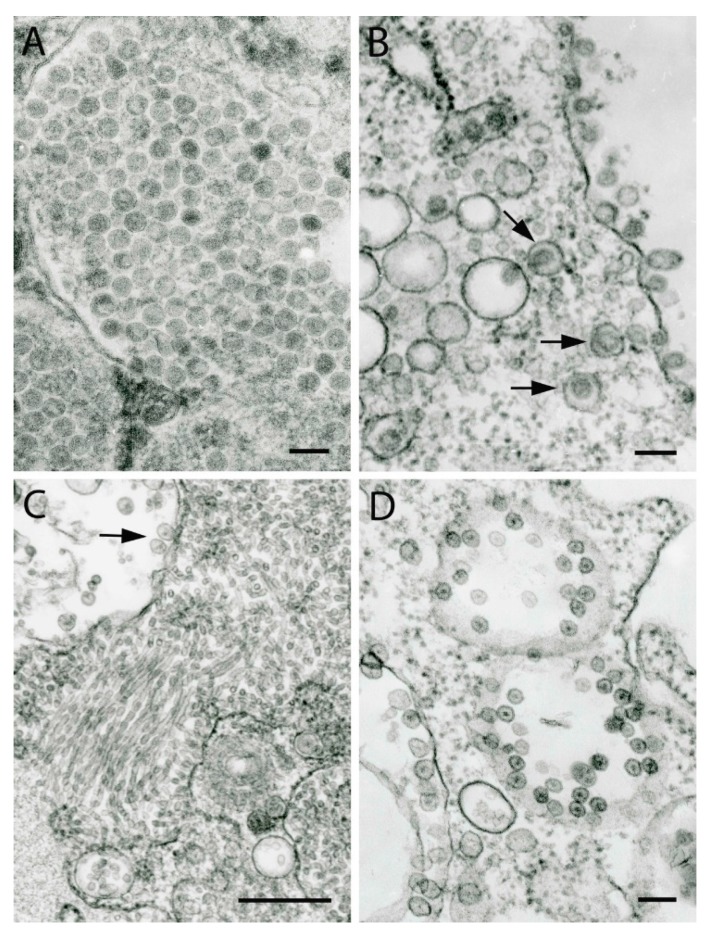
Ultrastructure of probable mesoniviruses and negeviruses. (**A**) Fragments of three large vacuoles in the cytoplasm of a C6/36 cell with virions 55 nm in diameter of Cavally virus (unclassified; probable genus *Alphamesonivirus*, family *Mesoniviridae*, order *Nidovirales*). Bar = 100 nm. (**B**) Virions (45-55 nm in diameter) of Bontag Baru virus (strain JKT-9891; unclassified, probable genus *Alphamesonivirus*, family *Mesoniviridae*, order *Nidovirales*), inside small cytoplasmic vacuoles (arrows) and at the surface of a C6/36 cell. Bar = 100 nm. (**C**) Fragment of a C6/36 cell infected with Negev virus (strain Houston-M30957-58; unclassified, proposed genus *Negevirus*), with enormous expansion of perinuclear space filled with tubules ~15 nm in diameter and of different lengths (up to 250 nm). The arrow indicates spherule-like structures ~65 nm in diameter in a vacuole at its periphery. Bar = 0.5 µm. (**D**) Three vacuoles with spherule-like structures 45 nm to 55 nm in diameter at inner periphery of their membranes in a C6/36 cell infected with Santana virus (strain BeAr 517449; unclassified; proposed genus *Negevirus*). Bar = 100 nm.

Alphavirus virions are icosahedral particles which appear spherical in ultrathin sections, 50-70 nm in diameter with dense nucleocapsid core (Figure 7C). Alphavirus replication complexes are associated with specific vesicles - spherules formed as invaginations of either plasma membrane (in vertebrate cells), [64] or limiting membranes of endosomes (occasionally in vertebrate but mostly in mosquito cells) [65,66]. These spherule-loaded endosomes (and/or lysosomes) are termed “type 1 cytopathic vacuoles” [67] and are very characteristic for alphavirus infection (Figure 7D) although recently they have been encountered in negevirus-infected C6/36 mosquito cells (Figure 8D). Alphavirus dense nucleocapsid cores ~40 nm in diameter can accumulate around cytopathic vacuoles [64] and can be found in the cytoplasm close to plasma membrane (Figure 7C, arrows). Alphavirus virions form by budding from the plasma membrane and can form paracrystalline arrays at the cell surface (Figure 7C). Electron microscopy was instrumental in the recent discovery of the insect-specific alphavirus, Eilat virus (Figure 10B,C), which cannot replicate in mammalian cells [68], allowing it to serve as a safe platform for vaccine development [69,70].

## 10. Mesoniviruses

Mesoniviruses were recently classified as a family [71,72] in the order *Nidovirales*, being distantly related to coronaviruses (family *Coronaviridae*, order *Nidovirales*). They appear to be very common and widespread in mosquito populations from different geographical and ecological locations [48]. Mature virions are spherical ~50 nm in diameter with a dense nucleocapsid core ~40 nm in diameter and surrounding envelope (Figure 8A). Usually they are found within large vacuoles often filling their entire space (Figure 8A) but can be localized in individual small vacuoles and at the cell surface (Figure 8B). Some infected cells had intravacuolar paracrystalline arrays consisting of empty and full virus particles but with less electron density than mature virions. At the periphery of these arrays, mature virions could be observed either free in cytosol or inside vacuoles [48].

## 11. Negeviruses

**Figure 9:**
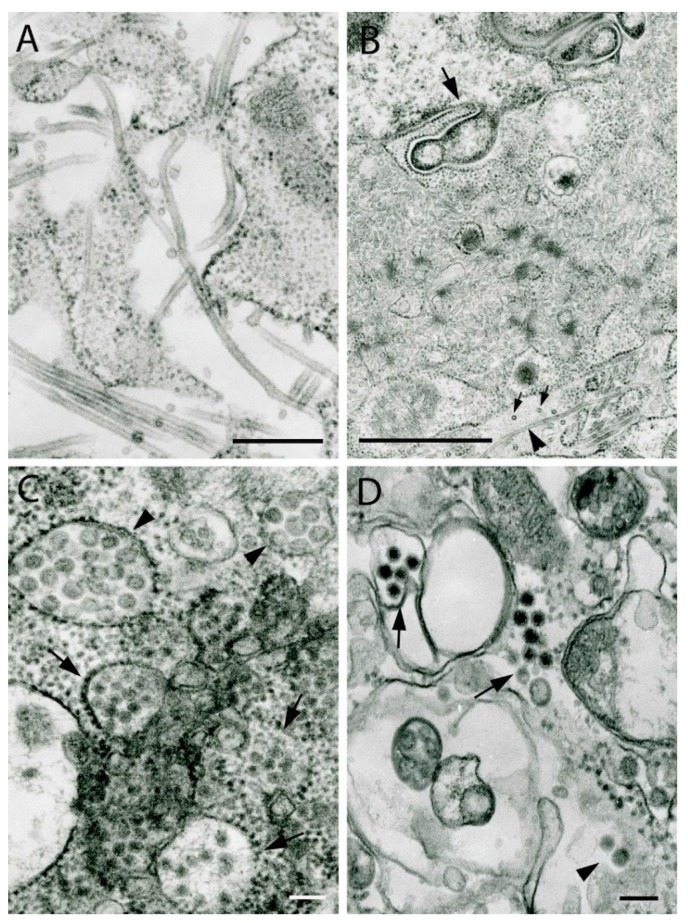
Ultrastructural cytopathology and mixed virus infections. (**A**) Enormous expansion of perinuclear space in a C6/36 cell infected with Loreto virus (strain 3940-83; unclassified, proposed genus *Negevirus*). Perinuclear space is filled with long tubules (up to several micrometers) ~30 nm in diameter. Bar = 0.5 µm. (**B**) Fragmentation of the cell nucleus (large arrow) and enormous expansion of perinuclear space-endoplasmic reticulum complex filled with tubules which can be very long (up to 1.3 um, arrowhead). Small arrows indicate cross-sections of the tubules ~25 nm in diameter. C6/36 cell is infected with BC 2-5 virus (unclassified; proposed genus *Negevirus*). Bar = 1 µm. (**C**) Mixed infection of a C6/36 cell with Karang Sari virus (strain JKT-10701; probable genus *Alphamesonivirus*) (arrowheads indicate virions 55 nm in diameter inside ER) and a flavivirus (arrows indicate virions 40 nm in diameter inside ER). Bar = 100 nm. (**D**) Mixed infection of a C6/36 cell with Kamphaeng Phet virus (strain KP84-0156; unclassified, probable genus *Alphamesonivirus*) (virions 55 nm in diameter indicated with an arrowhead) and a reovirus (45 nm in diameter, arrows). Bar = 100 nm.

**Figure 10:**
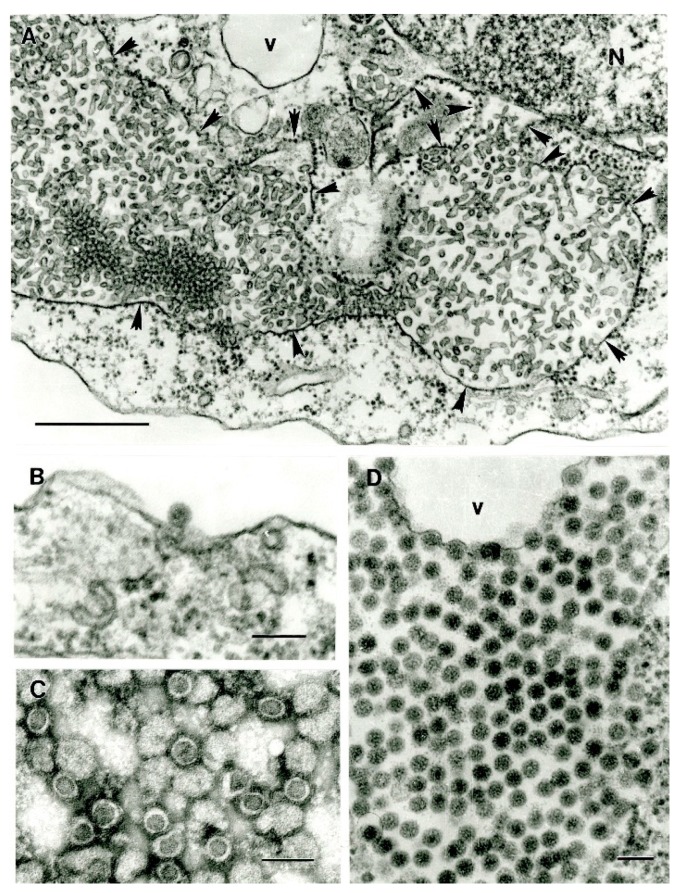
Ultrastructure of insect-specific viruses in insect cell cultures. (**A**) Negev virus (strain EO-239) in C6/36 cells induces enormous expansions of perinuclear space (indicated by arrowheads) filled with short tubules; N- cell nucleus, v – vacuole. Bar = 500 nm. (**B**) Eilat virus virion attached to the surface of C7/10 cell, 1 min post infection. Bar = 100 nm. (**C**) Purified virions of Eilat virus, negative staining with uranyl acetate. Bar = 100 nm. (**D**) Fragment of an intracytoplasmic inclusion of GMC-202 virus in C6/36 cell, presumably a novel *Entomobirnavirus*, isolated from mosquitoes collected on the Bolivar peninsula, Galveston county, Texas, U.S. Bar = 100 nm.

Insect-specific viruses isolated recently from mosquitoes and phlebotomine sandflies have been characterized and proposed to represent a new genus (*Negevirus*) related to genera of mite-infecting plant viruses (*Blunervirus*, *Cilevirus*, and *Higrevirus*) in the new family *Kitaviridae* [49,73], or novel members of *Entomobirnavirus*, family *Birnaviridae* (Figure 10D). They appear to be very common and widespread in insect populations on different continents as isolates have been obtained from pools of mosquitoes and sandflies collected in Israel, North and South America, Africa, and Indonesia. In negatively stained preparations of Piura virus, isolated from *Culex* sp. mosquitoes in Peru, spherical particles with diameters of ~45 nm and ~55 nm were found. The striking feature of negevirus infection of C6/36 mosquito cells are the cytopathic effects – enormous expansions of perinuclear space – a granular endoplasmic reticulum system which becomes filled with vesicles and/or microtubules (Figure 8C, Figure 9A,B andFigure 10A). They have a diameter of 20–25 nm but can be of different lengths. The shortest can resemble a rice grain in morphology and can be arranged in rosettes in cross-sections of ER. Others can be very long, reaching up to several micrometers (Figure 8C and Figure 9A,B). Expanded perinuclear space can occupy almost the whole cytoplasm of the cell. Some negeviruses cause long protrusions of the nucleus and deep invaginations of perinuclear membranes leading to the fragmentation of the nucleus (Figure 9B). Another peculiarity of negevirus infection of C6/36 cells is the formation of cytopathic vacuoles with spherule-like structures 45–55 nm in diameter at the inner periphery of their limiting membrane (Figure 8C,D). These vacuoles can reach 1.4 µm in diameter and are morphologically similar to type 1 cytopathic vacuoles in alphaviruses. They have been found in all 10 described negeviruses [49] and later in many others that have not yet been fully characterized. Their role in negevirus replication is not known.

## 12. Mixed Infections

Sometimes, after inoculation of C6/36 mosquito cells with homogenates of mosquito pools, several viruses can be observed in the culture, or even in the same cell. Figure 9C illustrates mixed infection with Karang Sari virus (genus *Alphamesonivirus*) (virions 55 nm in diameter inside ER cisterns) and an unknown flavivirus (smaller virions, 40 nm in diameter in different ER cisterns). Figure 9D demonstrates a co-infection with a Kamphaeng Phet virus (unclassified; probable genus *Alphamesonivirus*; virions 55 nm in diameter) and an unknown reovirus (virions 45 nm in diameter with a characteristic dark nucleocapsid core). We have also observed co-infection with Bontag Baru virus (unclassified; probable genus *Alphamesonivirus*) and an unknown flavivirus, Ngewontan (unclassified; proposed genus *Negevirus*) and Nam Dinh virus (genus *Alphamesonivirus*); Eilat virus (genus *Alphavirus*) (Figure 10B,C) and Negev virus (unclassified; proposed genus *Negevirus*) (Figure 10A). Three viruses have also been observed to infect the same culture: Bontag Baru virus (unclassified; probable genus *Alphamesonivirus*), an unknown flavivirus and an unknown rhabdovirus, the latter budding into an ER cistern occupied by a flavivirus virion. It is most likely that these viruses originate from different individual mosquitoes in the pool, but the possibility of a co-infection of one mosquito with several different viruses cannot be excluded.

## 13. Conclusions

The WRCEVA collection at the University of Texas Medical Branch in Galveston, Texas, plays an important role in virology research as a depository of natural viral biodiversity, and as a valuable resource for knowledge of viruses that are not only pathogenic for animals and humans but also are naturally occurring in arthropods. Propagation in cell cultures and subsequent examination of virus morphology by electron microscopy have been useful initial steps in the identification and characterization of novel viruses, giving indications for their further genetic characterization.

## References

[1] Murphy F.A. (2012). The Foundations of Virology - Discoverers and Discoveries, Inventors and Inventions, Developers and Technologies.

[2] Miller S.E., Lennette E.H., Smith T.F. (1999). Electron microscopy of viral infections. Laboratory Diagnosis of Viral Infections.

[3] Miller S.E., Lennette E.H., Lennette D.A., Lennette E.T. (1995). Diagnosis of viral infections by electron microscopy. Diagnostic Procedures for Viral, Rickettsial and Chlamydial Infections.

[4] Hsiung G.D., Fong C.K.Y., Landry M.L. (1994). Hsiung’s Diagnostic Virology as Illustrated by Light and Electron Microscopy.

[5] Adams J.R., Bonami J.R. (1991). Atlas of Invertebrate Viruses.

[6] Palmer E.L., Martin M.L. (1988). Electron Microscopy in Viral Diagnosis.

[7] Palmer E.L., Martin M.L. (1982). An Atlas of Mammalian Viruses.

[8] Doane F.W., Anderson N. (1987). Electron Microscopy in Diagnostic Virology: A Practical Guide and Atlas.

[9] Maramorosch K. (1978). The Atlas of Insect and Plant Viruses: Including Mycoplasmaviruses and Viroids.

[10] Dalton A.J., Haguenau F. (1973). Ultrastructure of Animal Viruses and Bacteriophages: An Atlas.

[11] Avakian A.A., Bykovsky A.F. (1970). Atlas of Anatomy and Ontogenesis of Human and Animal Viruses.

[12] Madeley C.R., Field A.M. (1988). Virus Morphology.

[13] Goldsmith C.S., Miller S.E. (2009). Modern uses of electron microscopy for detection of viruses. Clin. Microbiol. Rev..

[14] Goldsmith C.S., Ksiazek T.G., Rollin P.E., Comer J.A., Nicholson W.L., Peret T.C., Erdman D.D., Bellini W.J., Harcourt B.H., Rota P.A. (2013). Cell culture and electron microscopy for identifying viruses in diseases of unknown cause. Emerg. Infect. Dis..

[15] Goldsmith C.S. (2014). Morphologic differentiation of viruses beyond the family level. Viruses.

[16] Richert-Poggeler K.R., Franzke K., Hipp K., Kleespies R.G. (2018). Electron microscopy methods for virus diagnosis and high resolution analysis of viruses. Front. Microbiol..

[17] Mann B.R., McMullen A.R., Swetnam D.M., Salvato V., Reyna M., Guzman H., Bueno R., Dennett J.A., Tesh R.B., Barrett A.D. (2013). Continued evolution of west nile virus, houston, texas, USA, 2002–2012. Emerg. Infect. Dis..

[18] Dennett J.A., Bala A., Wuithiranyagool T., Randle Y., Sargent C.B., Guzman H., Siirin M., Hassan H.K., Reyna-Nava M., Unnasch T.R. (2007). Associations between two mosquito populations and west nile virus in harris county, texas, 2003–2006. J. Am. Mosq. Control Assoc..

[19] Meyer T.E., Bull L.M., Cain Holmes K., Pascua R.F., Travassos da Rosa A., Gutierrez C.R., Corbin T., Woodward J.L., Taylor J.P., Tesh R.B. (2007). West nile virus infection among the homeless, houston, texas. Emerg. Infect. Dis..

[20] Matsuno K., Weisend C., Kajihara M., Matysiak C., Williamson B.N., Simuunza M., Mweene A.S., Takada A., Tesh R.B., Ebihara H. (2015). Comprehensive molecular detection of tick-borne phleboviruses leads to the retrospective identification of taxonomically unassigned bunyaviruses and the discovery of a novel member of the genus *phlebovirus*. J. Virol..

[21] Matsuno K., Weisend C., Travassos da Rosa A.P., Anzick S.L., Dahlstrom E., Porcella S.F., Dorward D.W., Yu X.J., Tesh R.B., Ebihara H. (2013). Characterization of the bhanja serogroup viruses (*bunyaviridae*): A novel species of the genus *phlebovirus* and its relationship with other emerging tick-borne phleboviruses. J. Virol..

[22] Briese T., Chowdhary R., Travassos da Rosa A., Hutchison S.K., Popov V., Street C., Tesh R.B., Lipkin W.I. (2014). Upolu virus and aransas bay virus, two presumptive bunyaviruses, are novel members of the family *orthomyxoviridae*. J. Virol..

[23] Contreras-Gutierrez M.A., Nunes M.R.T., Guzman H., Uribe S., Suaza Vasco J.D., Cardoso J.F., Popov V.L., Widen S.G., Wood T.G., Vasilakis N. (2017). Sinu virus, a novel and divergent orthomyxovirus related to members of the genus thogotovirus isolated from mosquitoes in colombia. Virology.

[24] Lima J.A., Nunes Neto J.P., Castro K.S., Travassos da Rosa A.P.A., Tesh R., Nunes M.R.T., Popov V.L., Vasilakis N., Guzman H., Widen S. (2019). Characterization of triniti virus supports its reclassification in the family peribunyaviridae. J. Gen. Virol..

[25] Chiang J.O., Marciel de Souza W., Teixeira Nunes M.R., Acrani G.O., Paes de Andrade Travassos da Rosa A., Mesquita de Freitas N., Patroca da Silva S., Dorta da Silva P.H., Watanabe de Sousa A., Rodrigues S.G. (2018). Characterization of the gamboa virus serogroup (orthobunyavirus genus, peribunyaviridae family). Am. J. Trop. Med. Hyg..

[26] Hontz R.D., Guevara C., Halsey E.S., Silvas J., Santiago F.W., Widen S.G., Wood T.G., Casanova W., Vasilakis N., Watts D.M. (2015). Itaya virus, a novel orthobunyavirus associated with human febrile illness, peru. Emerg. Infect. Dis..

[27] Rogers M.B., Gulino K.M., Tesh R.B., Cui L., Fitch A., Unnasch T.R., Popov V.L., Travassos da Rosa A.P.A., Guzman H., Carrera J.P. (2017). Characterization of five unclassified orthobunyaviruses (*bunyaviridae*) from africa and the americas. J. Gen. Virol..

[28] Rogers M.B., Cui L., Fitch A., Popov V., Travassos da Rosa A.P., Vasilakis N., Tesh R.B., Ghedin E. (2014). Whole genome analysis of sierra nevada virus, a novel mononegavirus in the family *nyamiviridae*. Am. J. Trop. Med. Hyg..

[29] Auguste A.J., Kaelber J.T., Fokam E.B., Guzman H., Carrington C.V., Erasmus J.H., Kamgang B., Popov V.L., Jakana J., Liu X. (2015). A newly isolated reovirus has the simplest genomic and structural organization of any reovirus. J. Virol..

[30] Contreras-Gutierrez M.A., Guzman H., Cardoso J.F., Popov V.L., Nunes M.R.T., Uribe S., Widen S.G., Wood T.G., Vasilakis N., Tesh R.B. (2018). Genome sequence of chiqui virus, a novel reovirus isolated from mosquitoes collected in colombia. Microbiol. Resour. Announc..

[31] Forrester N.L., Widen S.G., Wood T.G., Travassos da Rosa A.P., Ksiazek T.G., Vasilakis N., Tesh R.B. (2013). Identification of a new newcastle disease virus isolate from indonesia represents an ancestral lineage of class ii genotype xiii. Virus Genes.

[32] Nasar F., Palacios G., Gorchakov R.V., Guzman H., Da Rosa A.P., Savji N., Popov V.L., Sherman M.B., Lipkin W.I., Tesh R.B. (2012). Eilat virus, a unique alphavirus with host range restricted to insects by rna replication. Proc. Natl. Acad. Sci. USA.

[33] Shi M., Lin X.D., Vasilakis N., Tian J.H., Li C.X., Chen L.J., Eastwood G., Diao X.N., Chen M.H., Chen X. (2016). Divergent viruses discovered in arthropods and vertebrates revise the evolutionary history of the *flaviviridae* and related viruses. J. Virol..

[34] Bolling B.G., Vasilakis N., Guzman H., Widen S.G., Wood T.G., Popov V.L., Thangamani S., Tesh R.B. (2015). Insect-specific viruses detected in laboratory mosquito colonies and their potential implications for experiments evaluating arbovirus vector competence. Am. J. Trop. Med. Hyg..

[35] Carrera J.P., Guzman H., Beltran D., Diaz Y., Lopez-Verges S., Torres-Cosme R., Popov V., Widen S.G., Wood T.G., Weaver S.C. (2015). Mercadeo virus: A novel mosquito-specific flavivirus from panama. Am. J. Trop. Med. Hyg..

[36] Guzman H., Contreras-Gutierrez M.A., Travassos da Rosa A.P.A., Nunes M.R.T., Cardoso J.F., Popov V.L., Young K.I., Savit C., Wood T.G., Widen S.G. (2018). Characterization of three new insect-specific flaviviruses: Their relationship to the mosquito-borne flavivirus pathogens. Am. J. Trop. Med. Hyg..

[37] Sadeghi M., Popov V., Guzman H., Phan T.G., Vasilakis N., Tesh R., Delwart E. (2017). Genomes of viral isolates derived from different mosquitos species. Virus Res..

[38] Blasdell K.R., Guzman H., Widen S.G., Firth C., Wood T.G., Holmes E.C., Tesh R.B., Vasilakis N., Walker P.J. (2015). Ledantevirus: A proposed new genus in the rhabdoviridae has a strong ecological association with bats. Am. J. Trop. Med. Hyg..

[39] Blasdell K.R., Widen S.G., Diviney S.M., Firth C., Wood T.G., Guzman H., Holmes E.C., Tesh R.B., Vasilakis N., Walker P.J. (2014). Koolpinyah and yata viruses: Two newly recognised ephemeroviruses from tropical regions of australia and africa. Vet. Microbiol..

[40] Contreras M.A., Eastwood G., Guzman H., Popov V., Savit C., Uribe S., Kramer L.D., Wood T.G., Widen S.G., Fish D. (2017). *Almendravirus*: A proposed new genus of rhabdoviruses isolated from mosquitoes in tropical regions of the americas. Am. J. Trop. Med. Hyg..

[41] Ghedin E., Rogers M.B., Widen S.G., Guzman H., Travassos da Rosa A.P., Wood T.G., Fitch A., Popov V., Holmes E.C., Walker P.J. (2013). Kolente virus, a rhabdovirus species isolated from ticks and bats in the republic of guinea. J. Gen. Virol..

[42] Palacios G., Forrester N.L., Savji N., Travassos da Rosa A.P., Guzman H., Detoy K., Popov V.L., Walker P.J., Lipkin W.I., Vasilakis N. (2013). Characterization of farmington virus, a novel virus from birds that is distantly related to members of the family *rhabdoviridae*. Virol. J..

[43] Vasilakis N., Castro-Llanos F., Widen S.G., Aguilar P.V., Guzman H., Guevara C., Fernandez R., Auguste A.J., Wood T.G., Popov V. (2014). Arboretum and puerto almendras viruses: Two novel rhabdoviruses isolated from mosquitoes in peru. J. Gen. Virol..

[44] Vasilakis N., Widen S., Mayer S.V., Seymour R., Wood T.G., Popov V., Guzman H., Travassos da Rosa A.P., Ghedin E., Holmes E.C. (2013). Niakha virus: A novel member of the family *rhabdoviridae* isolated from phlebotomine sandflies in senegal. Virology.

[45] Vasilakis N., Widen S., Travassos da Rosa A.P., Wood T.G., Walker P.J., Holmes E.C., Tesh R.B. (2013). Malpais spring virus is a new species in the genus *vesiculovirus*. Virol. J..

[46] Vasilakis N., Tesh R.B., Widen S.G., Mirchandani D., Walker P.J. (2019). Genomic characterisation of cuiaba and charleville viruses: Arboviruses (family rhabdoviridae, genus sripuvirus) infecting reptiles and amphibians. Virus Genes.

[47] Walker P.J., Firth C., Widen S.G., Blasdell K.R., Guzman H., Wood T.G., Paradkar P.N., Holmes E.C., Tesh R.B., Vasilakis N. (2015). Evolution of genome size and complexity in the *rhabdoviridae*. PLoS Pathog..

[48] Vasilakis N., Guzman H., Firth C., Forrester N.L., Widen S.G., Wood T.G., Rossi S.L., Ghedin E., Popov V., Blasdell K.R. (2014). Mesoniviruses are mosquito-specific viruses with extensive geographic distribution and host range. Virol. J..

[49] Vasilakis N., Forrester N.L., Palacios G., Nasar F., Savji N., Rossi S.L., Guzman H., Wood T.G., Popov V., Gorchakov R. (2013). Negevirus: A proposed new taxon of insect-specific viruses with wide geographic distribution. J. Virol..

[50] Nunes M.R.T., Contreras-Gutierrez M.A., Guzman H., Martins L.C., Barbirato M.F., Savit C., Balta V., Uribe S., Vivero R., Suaza J.D. (2017). Genetic characterization, molecular epidemiology, and phylogenetic relationships of insect-specific viruses in the taxon negevirus. Virology.

[51] Presti R.M., Zhao G., Beatty W.L., Mihindukulasuriya K.A., da Rosa A.P., Popov V.L., Tesh R.B., Virgin H.W., Wang D. (2009). Quaranfil, johnston atoll, and lake chad viruses are novel members of the family *orthomyxoviridae*. J. Virol..

[52] Da Silva E.V., Da Rosa A.P., Nunes M.R., Diniz J.A., Tesh R.B., Cruz A.C., Vieira C.M., Vasconcelos P.F. (2005). Araguari virus, a new member of the family *orthomyxoviridae*: Serologic, ultrastructural, and molecular characterization. Am. J. Trop. Med. Hyg..

[53] Bussetti A.V., Palacios G., Travassos da Rosa A., Savji N., Jain K., Guzman H., Hutchison S., Popov V.L., Tesh R.B., Lipkin W.I. (2012). Genomic and antigenic characterization of jos virus. J. Gen. Virol..

[54] Kosoy O.I., Lambert A.J., Hawkinson D.J., Pastula D.M., Goldsmith C.S., Hunt D.C., Staples J.E. (2015). Novel thogotovirus associated with febrile illness and death, united states, 2014. Emerg. Infect. Dis..

[55] Allison A.B., Ballard J.R., Tesh R.B., Brown J.D., Ruder M.G., Keel M.K., Munk B.A., Mickley R.M., Gibbs S.E., Travassos da Rosa A.P. (2015). Cyclic avian mass mortality in the northeastern united states is associated with a novel orthomyxovirus. J. Virol..

[56] Lvov D.K., Al’khovskii S.V., Shchelkanov M., Shchetinin A.M., Deriabin P.G., Aristova V.A., Gitel’man A.K., Samokhvalov E.I., Botikov A.G. (2014). Taxonomic status of the tyulek virus (tlkv) (*orthomyxoviridae*, *quaranjavirus*, quaranfil group) isolated from the ticks *argas vulgaris* filippova, 1961 (argasidae) from the birds burrow nest biotopes in the kyrgyzstan. Vopr. Virusol..

[57] Clerx J.P., Fuller F., Bishop D.H. (1983). Tick-borne viruses structurally similar to orthomyxoviruses. Virology.

[58] Zeller H.G., Karabatsos N., Calisher C.H., Digoutte J.P., Murphy F.A., Shope R.E. (1989). Electron microscopy and antigenic studies of uncharacterized viruses. I. Evidence suggesting the placement of viruses in families *arenaviridae*, *paramyxoviridae*, or *poxviridae*. Arch. Virol..

[59] Overby A.K., Popov V., Neve E.P., Pettersson R.F. (2006). Generation and analysis of infectious virus-like particles of uukuniemi virus (*bunyaviridae*): A useful system for studying bunyaviral packaging and budding. J. Virol..

[60] Westaway E.G., Mackenzie J.M., Kenney M.T., Jones M.K., Khromykh A.A. (1997). Ultrastructure of kunjin virus-infected cells: Colocalization of ns1 and ns3 with double-stranded rna, and of ns2b with ns3, in virus-induced membrane structures. J. Virol..

[61] Mackenzie J. (2005). Wrapping things up about virus RNA replication. Traffic.

[62] Miller S., Krijnse-Locker J. (2008). Modification of intracellular membrane structures for virus replication. Nat. Rev. Microbiol..

[63] Attoui H., Mohd Jaafar F., Biagini P., Cantaloube J.F., de Micco P., Murphy F.A., de Lamballerie X. (2002). Genus *coltivirus* (family *reoviridae*): Genomic and morphologic characterization of old world and new world viruses. Arch. Virol..

[64] Frolova E.I., Gorchakov R., Pereboeva L., Atasheva S., Frolov I. (2010). Functional sindbis virus replicative complexes are formed at the plasma membrane. J. Virol..

[65] Froshauer S., Kartenbeck J., Helenius A. (1988). Alphavirus RNA replicase is located on the cytoplasmic surface of endosomes and lysosomes. J. Cell Biol..

[66] Boehme K.W., Popov V.L., Heidner H.W. (2000). The host range phenotype displayed by a sindbis virus glycoprotein variant results from virion aggregation and retention on the surface of mosquito cells. J. Virol..

[67] Grimley P.M., Berezesky I.K., Friedman R.M. (1968). Cytoplasmic structures associated with an arbovirus infection: Loci of viral ribonucleic acid synthesis. J. Virol..

[68] Nasar F., Gorchakov R.V., Tesh R.B., Weaver S.C. (2015). Eilat virus host range restriction is present at multiple levels of the virus life cycle. J. Virol..

[69] Erasmus J.H., Seymour R.L., Kaelber J.T., Kim D.Y., Leal G., Sherman M.B., Frolov I., Chiu W., Weaver S.C., Nasar F. (2018). Novel insect-specific eilat virus-based chimeric vaccine candidates provide durable, mono- and multivalent, single-dose protection against lethal alphavirus challenge. J. Virol..

[70] Erasmus J.H., Auguste A.J., Kaelber J.T., Luo H., Rossi S.L., Fenton K., Leal G., Kim D.Y., Chiu W., Wang T. (2017). A chikungunya fever vaccine utilizing an insect-specific virus platform. Nat. Med..

[71] Zirkel F., Kurth A., Quan P.L., Briese T., Ellerbrok H., Pauli G., Leendertz F.H., Lipkin W.I., Ziebuhr J., Drosten C. (2011). An insect nidovirus emerging from a primary tropical rainforest. MBio.

[72] Zirkel F., Roth H., Kurth A., Drosten C., Ziebuhr J., Junglen S. (2013). Identification and characterization of genetically divergent members of the newly established family *mesoniviridae*. J. Virol..

[73] Kallies R., Kopp A., Zirkel F., Estrada A., Gillespie T.R., Drosten C., Junglen S. (2014). Genetic characterization of goutanap virus, a novel virus related to negeviruses, cileviruses and higreviruses. Viruses.

